# An Elementary Treatise on Human Anatomy

**Published:** 1889-07

**Authors:** 


**An Elementary Treatise on Human Anatomy.** By Joseph Leidy, M. D., Professor of Human and Comparative Anatomy and Zoology in the University of Pennsylvania; President of the Academy of Natural Sciences and of the Faculty of the Wagner Free Institute of Science, Philadelphia. Second edition; rewritten. With 495 illustrations. Octavo; pp. 950. Philadelphia: J. B. Lippincott Company. 1889. Buffalo: Peter Paul & Bro.

The first edition of Dr. Leidy's work on Human Anatomy has been long out of print, and it has been almost impossible to obtain a copy in any way. This was a matter of great regret, for we have always regarded it as the best text-book on the subject ever written. We use the superlative because nothing short of it will correctly express our opinion. It is, therefore, with great pleasure that we call attention to the second edition. After repeated solicitations, and after twenty-eight years, Dr. Leidy has prepared this edition. That all the author's students will receive it with enthusiasm we have no doubt, but all others interested in this important part of medical knowledge, and familiar with the former edition, will be glad to know that it can be again obtained.

The author need not excuse the appearance of another text-book on Anatomy, fearing that many will think it inferior and superfluous to the other excellent ones to be had; he rather ought to apologize for delaying the preparation of this edition for so many years.

Many features render this work distinctiveand superior. We can only refer to a few of them.

The nomenclature is generally in English, the Latin equivalent being given at the bottom of each page. The very bad tendency of giving names of persons to anatomical parts is, in great part, avoided, other and better terms being used. The description of the various parts, organs and tissue is simple, direct, and, at the same time, comprehensive. In this respect, the work deserves special praise. It is so written as to facilitate committing to memory the detail of anatomical study. This will be appreciated by students, and will simplify the work of teachers. For the other excellent features we must refer the reader to the work itself, and we feel sure that, upon examination, his opinion will coincide with ours.

The make-up of the book is a fine example of printers' art. The paper, printing, and illustrations are of superior quality. Of the last, nearly two hundred are original to this work, and are mostly by the author or by Dr. Schmidt. The publishers seem to have spared nothing in order to give the volume the setting it deserves. The result is in every way satisfactory.

